# The global burden of congenital heart disease

**DOI:** 10.5830/CVJA-2013-028

**Published:** 2013-06

**Authors:** Julien IE Hoffman

**Affiliations:** Department of Paediatrics, Cardiovascular Research Institute, University of California, San Francisco, California, USA

**Keywords:** fertility rate, per capita income, population age structure, cost disparities

## Abstract

**Abstract:**

Although the incidence of congenital heart disease (CHD) is similar worldwide, the burden of supporting these patients falls more heavily on countries with high fertility rates. In a country with a fertility rate of about eight per woman, the population has to support four times as many children with CHD as in a country with a fertility rate of two. Countries with the highest fertility rates tend to have the lowest incomes per capita, thus accentuating the disparity. Countries with high fertility rates have more children with congenital heart disease per wage earner. Improving local health services and controlling infectious diseases (diarrhoeal illness, rheumatic fever, measles, rotoviral infection) are important but are mere ‘band-aids’ compared to improving education, empowering women and reducing birth rates.

## Patients

Congenital heart disease, a major cause of serious morbidity and mortality, is common. It is usually defined as clinically significant structural heart disease present at birth;^1^ anomalies such as minor changes in the vena caval drainage that have no clinical importance are not included. Some caveats apply. For example, subaortic stenosis almost always develops well after birth but is included because it resembles other congenital forms of left ventricular outflow tract obstruction. Furthermore, some structural abnormalities that are genetically determined but usually present after infancy are not usually included in the totals; examples include the valve lesions of Marfan’s syndrome or obstruction due to hypertrophic cardiomyopathy. These genetic forms of heart disease are uncommon (Table 1) and including or excluding them from the totals makes little difference to the total incidence of congenital heart disease.

**Table 1:** Birth Incidence Of Different Forms Of Congenital Heart Disease

*Disease*	*Incidence/million live births*
Structural CHD	10 000–12 000
Bicuspid aortic valve	10 000–12 000
Genetic disorders	776
Dilated cardiomyopathy	6
Hypertrophic cardiomyopathy	50
Neuromuscular dystrophies	300
Connective tissue disorders: large countries, Marfan, mucopolysaccharidoses, etc	420

The incidence of congenital heart disease at birth (sometimes referred to as birth prevalence) depends on how a population is studied.^2^,^3^ Before the introduction of echocardiography, incidence figures ranged from five to eight per 1 000 live births but better diagnosis has detected many more with milder forms, so that current estimates range from eight to 12 per 1 000 live births. Much depends on how early and how intensively the diagnosis is made.

Performing echocardiograms on all neonates has shown that about 5% have small muscular ventricular septal defects, most of which close spontaneously before a year of age. Even more have small atrial septal defects that also close spontaneously. Many neonates have delayed closure of a patent ductus arteriosus. It therefore makes more sense to consider only lesions that need treatment in infancy or are present at a year of age. I think that the best current figure to use is 10–12 per 1 000 live births. Even though this includes some with small and mild defects, these patients still have significant cardiac murmurs, often come to medical attention, and cause much distress to families.

In addition to these, another 10–12 children per 1 000 live births have bicuspid aortic valves that are not stenotic. These seldom cause problems in childhood but account for a large number of adult patients who require treatment for late-onset aortic stenosis or regurgitation. Any consideration of the burden of congenital heart disease must take these into account. Table 1Table 1 shows some of these figures.

**Fig. 1.**
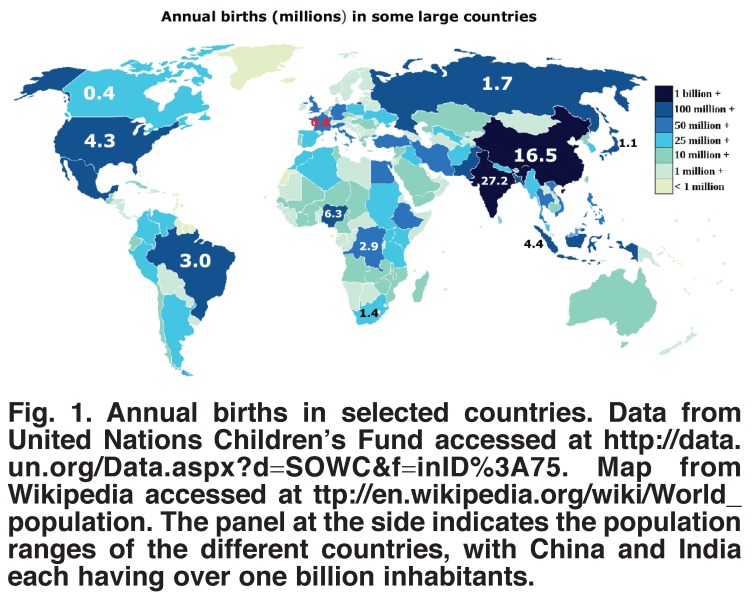
Annual births in selected countries. Data from United Nations Children’s Fund accessed at http://data.un.org/Data.aspx?d=SOWC<f=inID%3A75. Map from Wikipedia accessed at http://en.wikipedia.org/wiki/World_population. The panel at the side indicates the population ranges of the different countries, with China and India each having over one billion inhabitants.

**Fig. 2.**
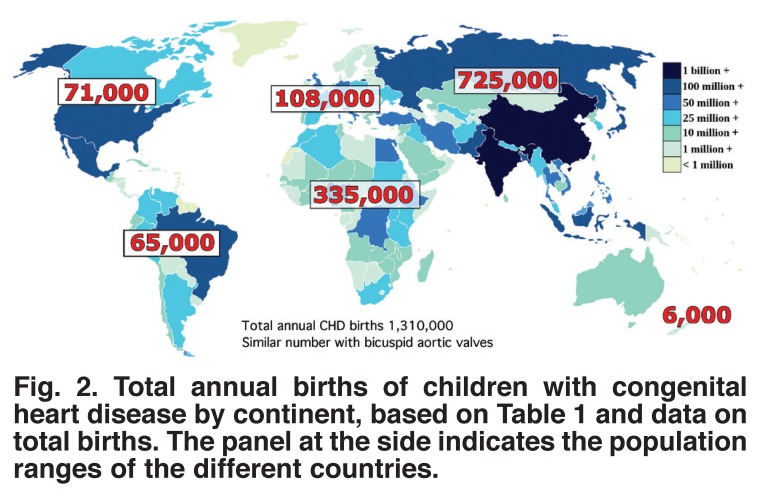
Total annual births of children with congenital heart disease by continent, based on Table 1 and data on total births. The panel at the side indicates the population ranges of the different countries.

As far as we can tell, the incidence of congenital heart disease is similar in all countries. There are some minor differences in types of congenital heart disease by country. For example, China and Japan have a higher incidence of subpulmonic ventricular septal defects, whereas coarctation of the aorta and aortic stenosis may be slightly less common in Asian countries. These variations do not appear to cause major differences in total incidence of congenital heart disease. Therefore, because we have good data on the annual number of births in different countries (Fig. 1), we can estimate how many children are born with congenital heart disease in different countries and continents (Fig. 2)

**Fig. 3.**
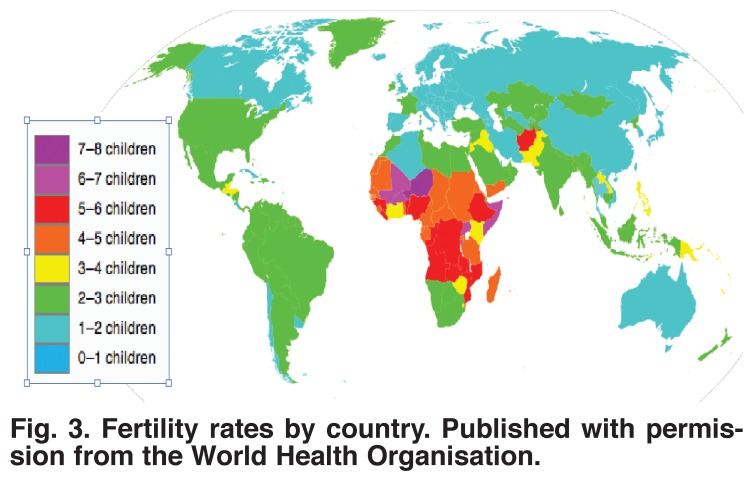
Fertility rates by country. Published with permission from the World Health Organisation.

The important datum is not how many children with congenital heart disease are born per country but how many of these children are born per million population, because this ratio tells us more about the burden of congenital heart disease. We can derive this information by knowing the fertility rate (the number of children born per woman) in different countries (Fig. 3). These data were taken from Wikipedia, based on data from the United Nations and the CIA World Factbook, accessed at http://en.wikipedia.org/wiki/List_of_sovereign_states_and_dependent_territories_by_fertility_rate.

**Fig. 4.**
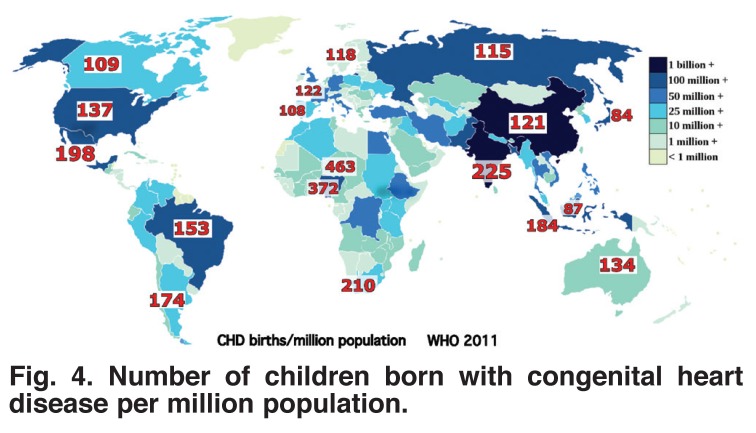
Number of children born with congenital heart disease per million population.

**Fig. 5.**
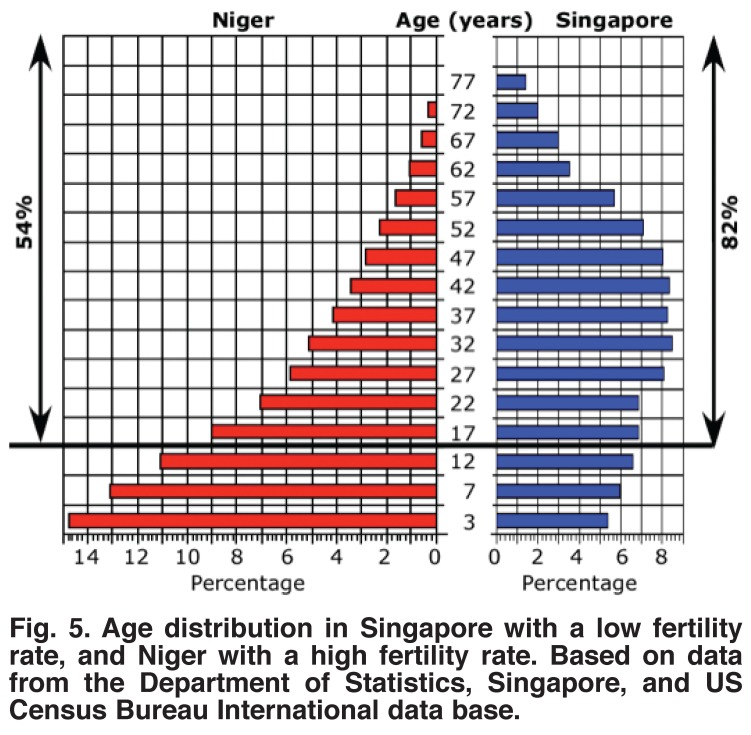
Age distribution in Singapore with a low fertility rate, and Niger with a high fertility rate. Based on data from the Department of Statistics, Singapore, and US Census Bureau International data base.

Because countries with higher fertility rates have more births per unit of population, they have a disproportionate number of children born with congenital heart disease (Fig. 4) and this imposes an added burden. This disproportionate burden on countries with high fertility rates is made even worse by the age structure of the population. As shown in Fig. 5, countries with high fertility rates have a higher proportion of younger people.

Because the burden of supporting children with congenital heart disease falls on those who are wage earners, it is reasonable to examine numbers of children with congenital heart disease per million wage earners. Therefore the 463 children with congenital heart disease per million population in Niger, where only 54% of the people are over 15 years of age, would be 463/0.54 = 857 per million wage earners. In Singapore, where 82% of the population is over 15 years of age, the ratio of children with congenital heart disease per million population is about 120. Not only does this disproportion in population ages increase the burden on countries with a high fertility rate, almost all of which are in sub-Saharan Africa, but the higher proportion of women of child-bearing age exacerbates their problems by leading to more future births of children with congenital heart disease.

In addition to having a higher proportion of children with congenital heart disease per wage earner, countries with high fertility rates tend to be those with lower per capita income. Therefore if we contrast two extremes of Niger and Singapore, we get the inequality shown in Table 2. This disparity is reduced somewhat by lower local costs in poorer countries. Costs such as labour to run hospitals, items constructed locally, food for patients, and fees paid to surgeons and anaesthetists, for example, are almost certainly much less in Niger than in Singapore. Other costs for imported goods, such as machines used for cardiopulmonary bypass, antibiotics and catheters used for interventional treatment are still very expensive and almost out of reach of the poorer countries.

**Table 2:** Estimates Of Potential Money To Spend On Children With CHD In Singapore And Niger. The GDP Values Are Corrected For Local Cost Of Living

*Country*	*CHD/million population*	*CHD/million wage earners*	*GDP per capita*	*GDP/CHD*
Singapore	~100	~120	$63 000	$525
Niger	~500	~850	$1 000	$0.85

Table 3 gives some representative costs in Africa, India and the United States. The International Children’s Heart Foundation sends teams to underdeveloped countries, and cites $2 500 for the cost of open-heart surgery; this is almost certainly subsidised.

**Table 3:** Representative Costs. Data Taken From Kumar And Balakrishnan^4^ And Pasquali Et Al.^5^

*Service*	*Africa*	*India*	*USA*
Open-heart surgery	US$10 000	$5 000	$12 000–50 000
128-slice CT angiogram	-	$350	$4 000
Colour Doppler echocardiography	$100	$30	$200

There are additional costs to congenital heart disease beyond surgical treatment: medical treatment, cost of transport to hospital, which is often difficult in rural Africa and Asia, and loss of parental working time when they have to take the children to a medical centre.^6^ These costs are disproportionately severe in countries with low incomes per capita.

In addition, both treated and untreated children with congenital heart disease are at risk of infective endocarditis. This risk may differ from country to country but we do not have good data. One current estimate by Knirsch and Nadal^7^ assessed the risk of congenital heart disease as 1.5 to six episodes per year per 100 000 adults, and 0.34–0.64 episodes per year per 100 000 children.

Infective endocarditis produces considerable morbidity, involves lengthy and costly treatment, and severely affects longevity. A follow-up study of patients with infective endocarditis and a variety of underlying heart diseases showed that fewer than 50% survived more than 20 years after the infection.^8^

Finally, children with congenital heart disease have more than heart disease to contend with. There can be associated defects in several other organ systems, and neurodevelopmental problems are particularly burdensome. Marino *et al.*^9^ reported in an American Heart Association scientific statement that moderate to severe neurodevelopmental disabilities occurred in over 50% of children with severe congenital heart disease or congenital heart disease palliated as neonates, and in 25% of those with less severe anomalies.

## Resources

**Fig. 6.**
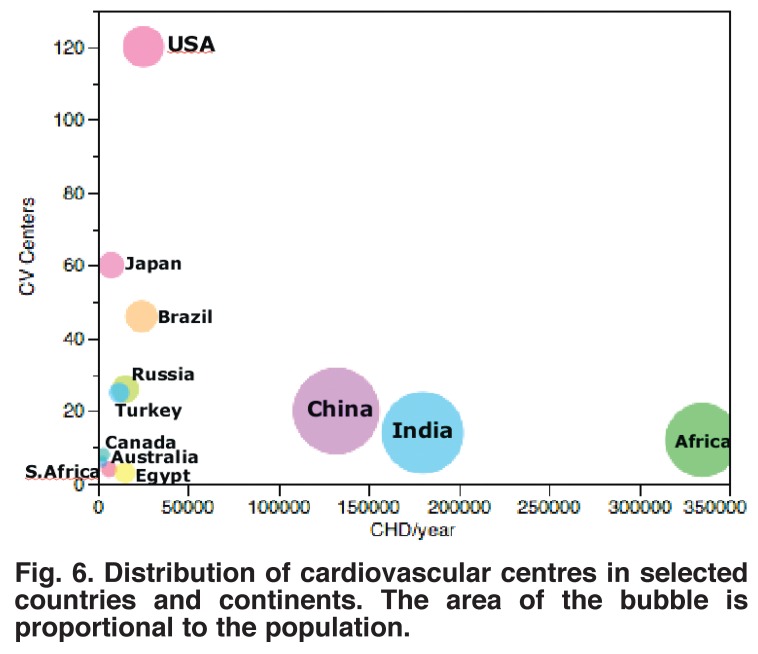
Distribution of cardiovascular centres in selected countries and continents. The area of the bubble is proportional to the population.

The resources to treat congenital heart disease are both inadequate and seriously maldistributed.^10^-^12^ The 2007–2009 World Society for Paediatric Heart Surgery Manpower Survey^11^ noted that about 75% of the world’s population have no access to cardiac surgery, and that the distribution of cardiac surgeons was very unbalanced (Table 4). This is in keeping with the variation in distribution of cardiovascular centres (Fig. 6).

**Table 4:** Ratio Of Cardiac Surgeons To Population On Different Continents

*Continent*	*Ratio cardiac surgeons:population*
North America	1:3.5 million
Europe	1:3.5 million
South America	1:6.5 million
Asia	1:25 million
Africa	1:38 million

## Prospects and potential solutions

The issues are so complex that it is difficult to know where to begin. Poverty, the greatest barrier to successful treatment of congenital heart disease, has multiple causes that are complex and difficult to remove. Natural resources may be inadequate or, where resources (oil, diamonds, ores) exist, corrupt and inefficient governance may prevent fair distribution. This is often compounded by limited education of the population, including its leaders, by unfair trade practices of the developed nations, and at times by well-meaning but ill-advised help from outside agencies.^13^-^15^ In addition, in sub-Saharan Africa, there is a huge deficit of all types of skilled medical personnel.^16^

Treatment of congenital heart disease by surgery or interventional cardiac catheterisation will always be relatively expensive, and expense will always be a major barrier to achieving good cardiac care of these children. This problem is not unique to congenital heart disease, and treatment of some non-cardiac diseases such as cancer, AIDS, drug-resistant tuberculosis and some chronic diseases may be as expensive. Unfortunately, attempts to alleviate poverty have had limited success. Nevertheless there are several strategies that can help to improve cardiac care. Because the specific problems may differ between countries, the mix of strategies may also need to be different.

Easing the burden of congenital heart disease can be divided into specific cardiac approaches that can be used on a near and mid-term time scale, and a more general socio-economic approach that will take much longer to implement. There are several models for the cardio-specific goals for treating congenital heart disease in underdeveloped countries, and they are discussed in detail by Hewitson and Zilia.^6^

The least appealing is to have the child and parents go where experts can treat the child optimally. This option is available to only a few privileged and wealthy families and does nothing to help the majority of people in the country. A second slightly more productive model is to have teams of doctors, nurses and technicians go to a country for a few weeks, and treat a set of patients who have been selected beforehand. This certainly benefits more children, but their follow up may be inadequate, the majority of children with congenital heart disease remain untreated, and this model does little to improve medical services in the country.

A better model would be to have these teams come to a country and help train local doctors, nurses and technicians, so that when the visitors leave, a functioning medical service is in place locally. This course, however, involves substantial investment by the host country that may be unwilling or unable to maintain this degree of sophisticated medical care. Developing locally low-cost substitutes for expensive imported products may reduce some of this disadvantage.

**Fig. 7.**
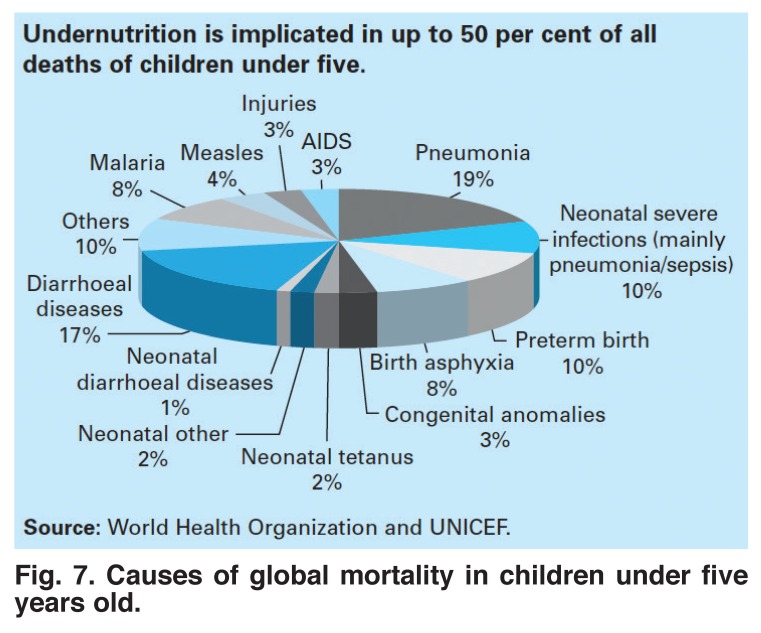
Causes of global mortality in children under five years old.

For long-term socio-economic goals, larger issues than treatment of congenital heart disease come into play. It is far from clear that children with congenital heart disease should get preferential use of scarce resources. In underdeveloped countries, congenital heart disease plays a minor role in child morbidity and mortality (Fig. 7).

**Fig. 8.**
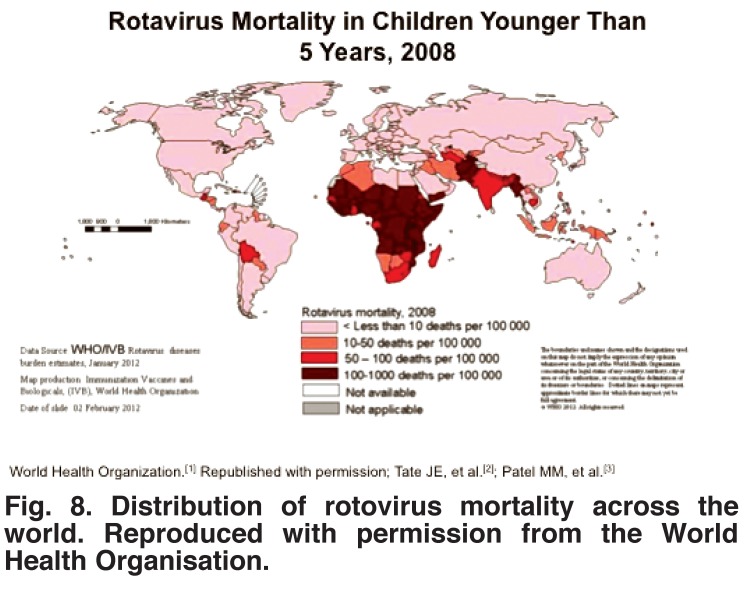
Distribution of rotovirus mortality across the world. Reproduced with permission from the World Health Organisation.

As shown in Fig. 7, congenital anomalies of all types account for only 5% of deaths, compared with 8% for malaria, 4% for measles, 17% for diarrhoeal diseases, and 29% for pneumonia and other infections, all of which are easier and cheaper to prevent or treat than are congenital heart diseases, and would be a better way to invest scarce resources. Fig. 8 shows, for example, the enormous death rate from rotovirus disease, for which an excellent and low-cost vaccine is now available but not yet extensively used.

Finally, in this list of preventable diseases, we should mention rheumatic heart disease. Although its prevalence is not well defined, it probably affects as many patients as do all forms of congenital heart disease, including bicuspid aortic valves. Inasmuch as rheumatic heart disease usually follows repeated episodes of acute rheumatic fever, and that treatment of acute streptococcal pharyngitis with penicillin is cheap and effective, this would be an excellent field into which to put scarce resources.^6^

**Fig. 9.**
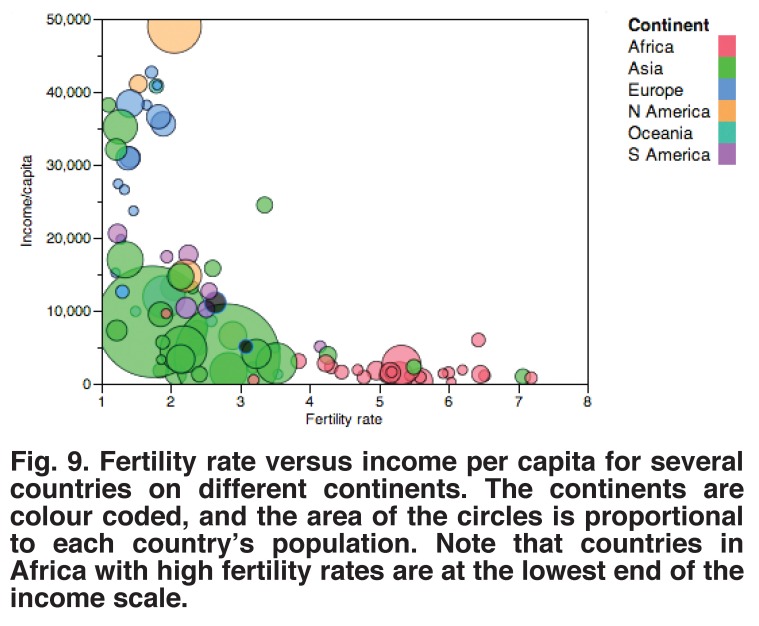
Fertility rate versus income per capita for several countries on different continents. The continents are colour coded, and the area of the circles is proportional to each country’s population. Note that countries in Africa with high fertility rates are at the lowest end of the income scale.

**Fig. 10:**
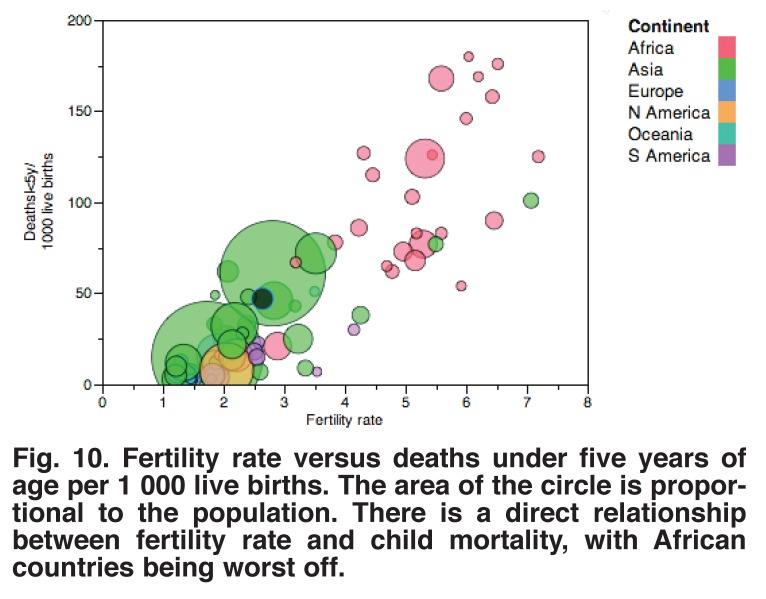
Fertility rate versus deaths under five years of age per 1 000 live births. The area of the circle is proportional to the population. There is a direct relationship between fertility rate and child mortality, with African countries being worst off.

Treating congenital heart disease, however, is always going to be expensive, and it may be worth giving more thought to its prevention. An approach that has been shown to be effective in a variety of countries is to educate women and improve efforts for family planning. The relationships between excessive fertility and income (Fig. 9) and child mortality (Fig. 10) suggest that reduction in fertility would be more effective than any other intervention we could make at present.

For example, if it were possible to reduce fertility in Niger from eight to four children per woman, the incidence of congenital heart disease would be reduced by one-half, and if each woman had only two children, the incidence would be reduced by 75%. This is a dramatically greater reduction than possible with any form of treatment; it comes at little cost to the medical services and has the added benefit that it may be associated with an increased per capita income.^16^

In Mali, a country with a very high fertility rate (before the coup), with the cooperation of imams who understood the problems, the fertility rate dropped from 7.29 in 2009 to 6.35 in 2012. Once such a change starts, the gains accumulate so that there is more money available to treat congenital heart disease in the remaining children.

Reductions in fertility rate with improvement in the national economy have been demonstrated in China, Vietnam and many other countries, even though multiple factors were probably involved. Hans Rosling, an epidemiologist who has worked in this field for many years, made a superb YouTube presentation that demonstrates these changes.^17^ It is well worth looking at it.

Improvement in treating congenital heart disease is a long-range enterprise. In keeping with the notion that preventing disease is often cheaper than treating it, reducing the fertility rate is perhaps the most important approach and one that has many benefits beyond reducing the incidence of the disease. Second, investing resources in preventing or treating diseases such as malnutrition, malaria, diarrhoea, measles, pneumonia and rheumatic fever would greatly reduce the child mortality rate, and would then free up money for other purposes, such as establishing regional centres for treating congenital heart disease.

One way of achieving these goals with the least expenditure might be for governments in Africa and Asia to invest in local clinical centres where people have access to health education, diagnosis and treatment for common illnesses. Experience in countries such as China and the former southern Rhodesia, now Zimbabwe, have shown that it is possible to train nurses or nurses’ aides for relatively short periods and have them work in local centres where they can diagnose and treat the simple common illnesses. If this were done after consulting the local population and their leaders, whether these be chieftains, sangomas, ngangas, shamans or imams, this would secure their cooperation and reduce resistance from the population to a government structure imposed on them.

## Conclusion

The burden of congenital heart disease falls most heavily on countries with the lowest incomes and the highest fertility rates. Strategies for improvement include preventing excessive numbers of births of children with congenital heart disease by lowering the fertility rate. Money for treating chronic diseases, such as congenital heart disease is always in short supply but can be freed up if more easily preventable acute diseases, such as malnutrition, measles, rotavirus infections or rheumatic fever are made the targets for concerted action. Finally, establishing local health centres with the support of the local population may make it easier to give advice about health and nutrition, administer vaccines and treat streptococcal infections early.
